# Successful Multimodal Therapeutic Approach for CD30‐positive Folliculotropic Mycosis Fungoides

**DOI:** 10.1111/ddg.70172x

**Published:** 2026-03-06

**Authors:** Chiara L. Blomen, Inga Hansen‐Abeck, Andrea Baehr, Stefan W. Schneider, Christoffer Gebhardt, Nina Booken

**Affiliations:** ^1^ Klinik und Poliklinik für Dermatologie und Venerologie, Universitätsklinikum Hamburg‐Eppendorf Hamburg Germany; ^2^ Klinik für Strahlentherapie und Radioonkologie, Universitätsklinikum Hamburg‐Eppendorf Hamburg Germany

Dear Editors,

Folliculotropic mycosis fungoides (FMF) is a rare and distinct variant of mycosis fungoides with perifollicular infiltration of CD4‐positive T cells, accompanied by follicular mucinosis usually without epidermotropism. A distinction is made between early and advanced stages of the disease, which differ in clinical presentation and prognosis.^1^ FMF typically occurs in the head and neck area and on the trunk. Clinically, the early stage is characterized by erythematous patches, which may be accompanied by alopecia, acneiform exanthema including comedones, and miliaria or keratosis pilaris‐like lesions.^2^ In the advanced stage, plaques or tumors are present, analogous to classic mycosis fungoides with a poorer prognosis.^1^

In cases with CD30 positivity, differentiation from lymphomatoid papulosis (LyP) and primary cutaneous CD30‐positive large anaplastic T‐cell lymphoma (cALCL) is relevant, but only possible through clinical‐pathological correlation.^3^ LyP is clinically characterized by complete healing of the papulonodular lesions within a few weeks.^4^ Solitary, often ulcerated lesions are associated with cALCL, while FMF is characterized by the simultaneous occurrence of patches, plaques and possibly tumors.

The treatment of FMF depends on the stage of the disease and can be challenging,^2^ which is why it is particularly important to consider synergistically effective, multimodal treatment concepts. In addition to topical, UV and radiotherapeutic approaches, systemic first‐line therapies such as bexarotene, interferon‐alpha and methotrexate or, in second‐line therapy, brentuximab vedotin (BV) and mogamulizumab can be used.^5^, ^6^, ^7^ BV is an antibody against CD30 that is conjugated with the antimicrotubular agent monomethyl auristatin E (MMAE). After binding to the CD30‐positive T cell and internalization, MMAE exerts its apoptotic effect by destroying the microtubules. BV acts as a radiosensitizer in tumor cells by increasing DNA double‐strand breaks.^8^, ^9^ Almost complete healing of the skin lesions of advanced cutaneous T‐cell lymphoma has been described after the combination of ultra‐hypofractionated low‐dose whole skin irradiation and brentuximab vedotin.^9^

A 64‐year‐old male patient presented with an exophytic tumor on the scalp that had been growing for a year, accompanied by erythematous plaques and an acneiform exanthema on the trunk (Figure 1). Further complaints as well as regular medication intake were denied.

**FIGURE 1:**
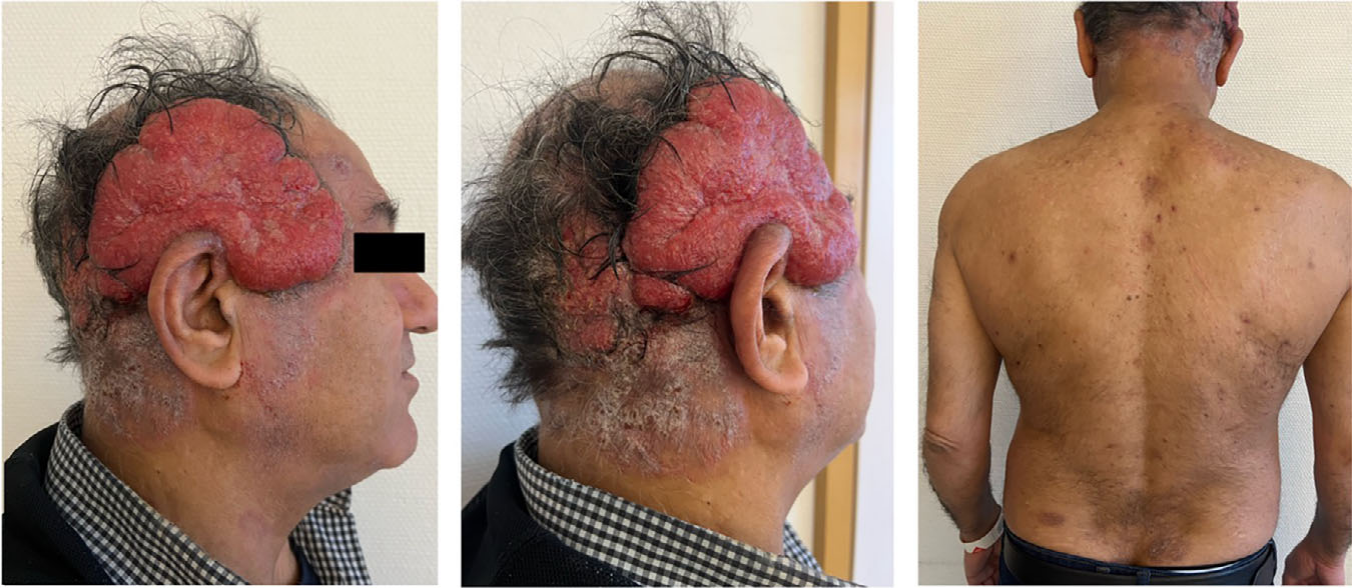
Initial presentation: tumor on the scalp; plaques and acneiform exanthema on the trunk.

Skin biopsies showed pleomorphic, folliculotropic T‐cell proliferation with CD30‐positive large cell transformation. 15–20% CD30‐positive T cells were found in the plaque portion and 40% in the nodular portion. Due to cervical lymphadenopathy, a lymph node biopsy was performed to rule out nodal involvement. Further tests enabled blood and organ involvement to be ruled out (using FACS analysis, computer tomography from neck to pelvis and magnetic resonance imaging of the head). Based on clinical‐pathological correlation, the diagnosis of a CD30‐positive FMF (tumor classification according to ISCL/EORTC T3 N1a M0 B0, stage IIB) was made.

Based on the recommendations of the interdisciplinary tumor board, methotrexate 15 mg subcutaneous and folic acid once a week for three weeks as well as antibiotic therapy based on the Lindahl regimen were initially administered.^10^ The rationale behind antibiotic therapy for cutaneous T‐cell lymphomas is the tumor‐associated skin barrier disorder and the associated increased *Staphylococcus aureus* colonization. This not only leads to increased bacteremia and thus increased morbidity and mortality, but also to increased release of enterotoxins. These act as superantigens for T cells and can contribute to tumor progression of cutaneous T‐cell lymphomas.^10^

Due to inadequate treatment response and gastrointestinal side effects with methotrexate and high CD30 positivity, the patient was switched after three weeks to a second‐line combination therapy with BV at standard dose (1.8 mg/kg BW) and ultra‐hypofractionated local radiotherapy.^11^ After just two cycles of BV and application of local irradiation to the capillitium using 8 Gy in two fractions of 4 Gy each, a significant improvement in the clinical picture was seen. After five cycles of BV therapy, a grade II peripheral polyneuropathy (PNP) according to CTCAE criteria arose, and as a result the dosage was reduced to 1.2 mg/kg BW. After a total of seven BV cycles, the residual tumor was excised, revealing extensive inflammatory scarring changes and only isolated residual lymphoma cells. Subsequently, a split‐thickness skin graft was performed to cover the defect and bexarotene 150 mg/m^2^ per day was administered as maintenance therapy (Figure 2). After six months, there was no evidence of recurrence on the scalp, with residual cutaneous manifestations on the trunk.

**FIGURE 2:**
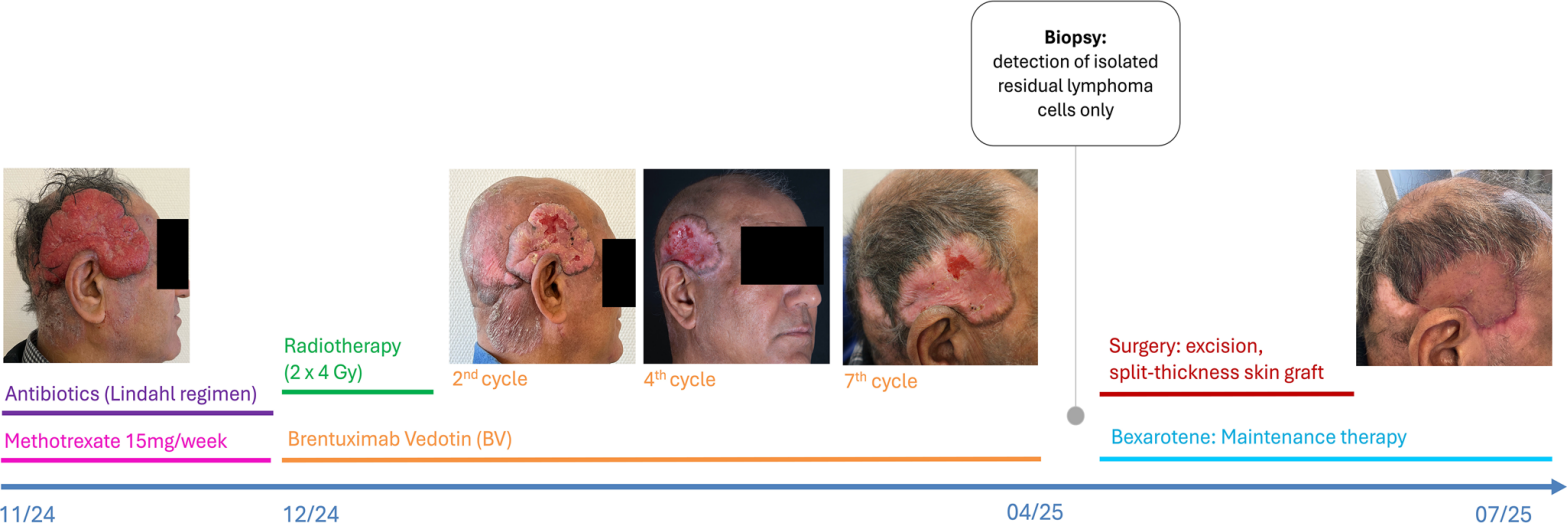
Timeline of therapy and clinical response.

This case emphasizes the importance of carefully distinguishing CD30‐positive FMF from cALCL and LyP in the differential diagnosis. The correct diagnosis can be made by clinical‐pathological correlation. The treatment of FMF depends on the stage of the disease. The combination of BV and ultra‐hypofractionated radiotherapy can produce excellent clinical results and enable a significant reduction in tumor volume, particularly in cases with high CD30 expression.

## CONFLICT OF INTEREST STATEMENT

Nina Booken received honoraria from Takeda GmbH & Co. KG.
